# Addition of Salvage Immunotherapy to Targeted Therapy in Patients with Metastatic Renal Cell Carcinoma

**DOI:** 10.3390/curroncol28060421

**Published:** 2021-11-30

**Authors:** Scott J. Dawsey, Moshe C. Ornstein

**Affiliations:** Department of Hematology & Medical Oncology, Cleveland Clinic Taussig Cancer Institute, Cleveland, OH 44195, USA; dawseys@ccf.org

**Keywords:** renal cell carcinoma, kidney cancer, salvage therapy, targeted therapy, immunotherapy, checkpoint inhibitors, VEGF TKI

## Abstract

There have been significant advances in the treatment of metastatic renal cell carcinoma (mRCC), with immunotherapy (IO)-based combinations as the standard-of-care treatment in the front-line setting. IO in this setting is paired with another IO agent or with a vascular endothelial growth factor receptor (VEGF-R) tyrosine kinase inhibitor (TKI). One IO/IO combination and four IO/TKI combinations are currently approved. However, the role of the salvage IO in patients with disease progression on TKI monotherapy is uncertain. Here, we present a case series of five patients who were on single-agent TKI therapy for treatment-refractory mRCC and upon disease progression had an IO agent added to their TKI. The median duration of TKI monotherapy was 11.2 months (range, 1.7–31.1 months), and the median duration of response after the addition of IO was 4 months (range, 2.8–10.5 months). Although IO salvage therapy has a plausible rationale, this case series did not show a clear benefit to this approach. Further clinical trials are needed to determine the clinical utility of IO salvage therapy in mRCC.

## 1. Introduction

The standard-of-care first-line treatment for metastatic renal cell carcinoma (mRCC) has changed substantially over the last few decades. The era of targeted therapy saw an emergence of agents targeting the vascular endothelial growth factor receptor (VEGF-R) pathway, including sunitinib [1], axitinib [2], pazopanib [3], sorafenib [4], bevacizumab [5,6], cabozantinib [7] and tivozanib [8]; as well as the mammalian target of rapamycin (mTOR) inhibitors everolimus [9] and temsirolimus [10]. The subsequent immunotherapy (IO) era began with the approval of nivolumab for refractory mRCC [11] followed by a host of IO-based combinations that now serve as the standard of care for treatment-naïve mRCC. These IO-based combinations are axitinib plus pembrolizumab [12], cabozantinib plus nivolumab [13], ipilimumab plus nivolumab [14], and lenvatinib plus pembrolizumab [15]. These combinations have all demonstrated a survival benefit when compared with the prior standard of care treatment in this setting.

With the approval of multiple IO-based combination therapies for treatment naïve mRCC, new questions have arisen for subsequent lines of treatment. One important unanswered question is whether patients on single-agent IO or TKI can benefit from a salvage approach in which the complimentary agent is added at the time of disease progression (PD). There is data demonstrating that the addition of salvage ipilimumab to patients who have PD on nivolumab monotherapy does not produce the same efficacy as when ipilimumab plus nivolumab are given in combination from the onset [16,17,18]. However, data for salvage IO after PD on TKI is lacking.

Based on CheckMate-025, single-agent IO with nivolumab has demonstrated a significant objective response rate (ORR) (25% vs. 5%; odds ratio, 5.98 [95% CI, 3.68 to 9.72]; *p* < 0.001) and median overall survival (25 months vs. 19.6 months; hazard ratio of death was 0.73 (98.5% CI, 0.57 to 0.93; *p* = 0.002)) benefit compared with everolimus in patients whose mRCC progressed on antiangiogenic therapy [11]. Although this demonstrates IO activity post-TKI, data is lacking regarding the *addition* of salvage IO for a patient who develops PD on TKI monotherapy. Herein, we present five cases of patients with mRCC on TKI monotherapy who received salvage IO therapy upon PD.

## 2. Results

Under a Cleveland Clinic Foundation Institutional Review Board (IRB)-approved retrospective protocol (IRB-19-609, approval date 5 January 2019), five patients with mRCC treated with TKI and subsequent salvage IO therapy were identified. Key features are highlighted in Table 1. In total, four (80%) were male, and median age at diagnosis was 57 (range, 44–66). All patients had clear cell RCC histology. Four (80%) of the patients had undergone a prior nephrectomy. All patients had intermediate-risk RCC per IMDC criteria (though IMDC risk was unknown for one patient).

**Figure 1:**
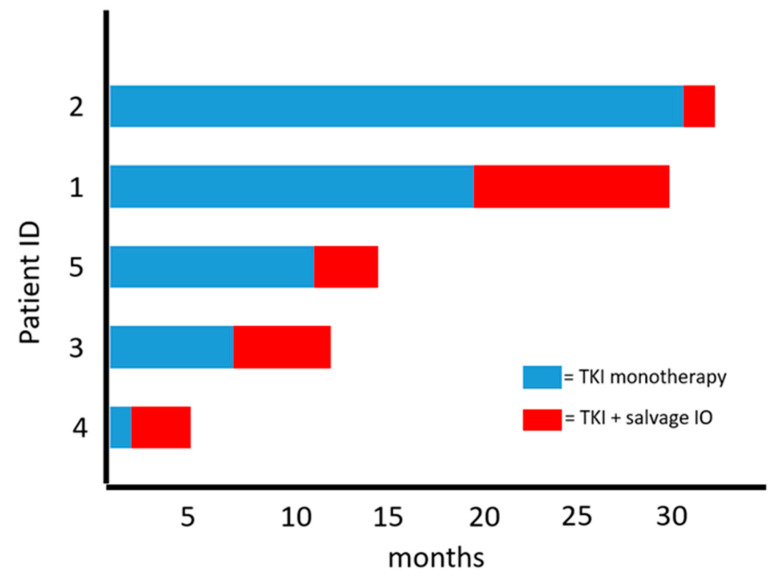
Duration on TKI monotherapy and with TKI and salvage IO.

The median number of treatments prior to the TKI for which salvage IO was added was three (range, 2–3). All patients had received at least one IO and TKI either alone or in combination as a prior therapy (Table 1). The TKI immediately prior to salvage IO was axitinib (*n* = 3) or cabozantinib (*n* = 2). Median duration of therapy on TKI monotherapy was 11.2 months (range, 1.7–31.1 months). Patients on axitinib had pembrolizumab added as the salvage IO therapy to align with KEYNOTE-426 [12], and patients on cabozantinib received the addition of salvage nivolumab per CheckMate-9ER [19]. The best response to salvage IO therapy was SD in three patients (60%) and PD in two patients (40%). The median duration of response after salvage IO was 4 months (range, 2.8–10.5 months) (Table 1; Figure 1).

## 3. Discussion

In this small case series of heavily pretreated patients with mRCC who had disease progression (PD) while on TKI monotherapy, the addition of salvage IO did not appear to provide additional significant benefit beyond what has been seen with single-agent monotherapy [11,20]. In total, the median duration of therapy while on TKI monotherapy was 11.2 months (range, 1.7–31.1 months) and the median duration of therapy while on TKI with salvage IO was only 4 months (range, 2.8–10.5 months) (Figure 1). A primary limitation to the case series (in addition to a small sample size) is that all patients had received a prior IO agent at some point during their treatment course (Table 1). As such, it is plausible that these patients were already IO-resistant to some degree and were therefore unlikely to obtain a clinical benefit from salvage IO therapy.

Although the IO/IO combination of ipilimumab and nivolumab is established as a standard of care for IMDC intermediate and poor risk mRCC, it comes with a significant risk of immune-related adverse events (IRAEs). Therefore, several studies have evaluated the utility of single-agent nivolumab followed by the addition of ipilimumab in order to improve tolerability. In the TITAN-RCC trial, patients with mRCC were treated with nivolumab monotherapy, and those who did not achieve a response as well as those who developed PD received a “boost” with ipilimumab/nivolumab. Included in this trial were 98 patients who received nivolumab as monotherapy following disease progression on TKI. Confirmed objective response rate (ORR) for this population was 17% with nivolumab monotherapy alone. The confirmed ORR in TKI-pretreated mRCC who had PD on TKI and then received a nivolumab/ipilimumab “boost” was 21% [21]. These data suggest modest efficacy with nivolumab monotherapy following PD on TKI and some response improvement with an ipilimumab/nivolumab salvage approach.

Similarly, the OMNIVORE trial investigated the role of nivolumab monotherapy with the addition of ipilimumab for patients with PD or stable disease (SD) at 6 months. Of 57 patients who met these criteria for salvage ipilimumab, only 2 patients (4%) converted to partial response (PR) and 0 patients converted to complete response (CR) [16]. Similar data from the HCRN GU16-260 trial showed that of 27 patients who received IO/IO after single-agent IO, 11% achieved PR and 0% achieved CR [17]. In a study of 45 patients with mRCC who received at least one dose of salvage IO/IO, the ORR was 20% with no CR [22]. Taken together, these data demonstrate the efficacy of single-agent nivolumab, but that the addition of salvage ipilimumab does not reproduce the same clinical responses seen when the combination is given upfront.

While the aforementioned trials highlight the relative lack of efficacy with salvage ipilimumab in patients receiving nivolumab, there is no significant data about the role of salvage IO for patients treated with TKI monotherapy. In general, the approach to the patient who has disease progression while on a either a TKI or an IO agent is to discontinue the current agent and switch to a subsequent therapy based on a host of data regarding the efficacy of TKI following IO, and vice versa [11,23,24,25]. However, the question remains of whether there is a benefit to the addition of IO after PD while on TKI, and vice versa.

There is some rationale to the role of maintaining TKI therapy and adding salvage IO. It is well-established that the tumor microenvironment (TME) contains certain immunosuppressive factors such as regulatory T cells (Tregs) and myeloid-derived suppressor cells (MDSCs). These immunosuppressive components are decreased with VEGF-R inhibition, therefore creating a more immune permissive TME, which is more likely to respond to IO-based therapy [26,27]. Given these facts, the continued presence of a VEGF-R TKI therapy while adding an IO agent has biological rationale. A similar hypothesis can be generated for the addition of TKI therapy in a patient who develops disease progression while on single-agent IO. However, despite scientific plausibility, the standard of care is to switch therapy upon disease progression on TKI monotherapy, and not to add a salvage IO agent. Additional clinical trials are needed to define the role of salvage therapy in mRCC. Though not an exact comparison, the PEDIGREE trial (NCT03793166) will shed some light on this clinical question. In this trial, patients with mRCC who have a PR of SD following treatment with ipilimumab and nivolumab will be randomized to receive either nivolumab alone or nivolumab in combination the VEGF-R TKI cabozantinib. While this may not directly answer the role of salvage IO in the setting of PD while on TKI, it will provide some evidence as to the role of such combinations vs. monotherapy in a SD/PR patient population.

There are clear challenges to developing a clinical trial of salvage IO. Given the aforementioned data of the poor efficacy of salvage ipilimumab, there will be reasonable hesitance to such an approach. In the setting of multiple approved treatment options in mRCC, trials that investigate the role of IO vs. IO/TKI in patients who develop PD on IO-based therapy are ongoing (NCT04338269). Strategies that investigate salvage approaches remain worthy of consideration.

## 4. Conclusions

Despite significant advanced in the management of mRCC, a number of questions still remain. Primary among these questions is the role of the addition of salvage therapy at the time of disease progression vs. simply switching to a new therapy. In this small series, there was no clear benefit to the addition of salvage IO therapy in patients with disease progression on TKI. Further trials are needed to define treatment sequencing and the role of salvage therapy in mRCC.

## Figures and Tables

**Table 1:** Patient and treatment characteristics.

Patient	Age	Histology	Prior Therapies	TKI Monotherapy and Duration Prior to PD (months)	Best Response on TKI Monotherapy	Addition of IO Salvage Therapy and Duration (months)	Salvage Therapy Best Response
1	45	ccRCC with sarcomatoid features	Pazopanib; Cabozantinib; Nivolumab	Axitinib (19.4)	SD	Pembrolizumab (10.5)	SD
2	66	ccRCC with sarcomatoid features	Pazopanib; Nivolumab	Axitinib (31.1)	SD	Pembrolizumab (2.8)	PD
3	57	ccRCC	Atezolizumab; Atezolizumab & Bevacizumab; Axitinib	Cabozantinib (7.1)	SD	Nivolumab (5.6)	SD
4	44	ccRCC	Sunitinib; Ipilimumab & Nivolumab; Cabozantinib	Axitinib (1.7)	SD	Pembrolizumab (4.0)	SD
5	66	ccRCC	Axitinib & Pembrolizumab	Cabozantinib (11.2)	SD	Nivolumab (3.7)	PD

## Data Availability

Data available on request due to privacy restrictions. The data presented in this study are available on request from the corresponding author. The data are not publicly available due to being part of the electronic medical record.
